# Antioxidant Activity of Synthetic Polymers of Phenolic Compounds

**DOI:** 10.3390/polym12081646

**Published:** 2020-07-24

**Authors:** Subhalakshmi Nagarajan, Ramaswamy Nagarajan, Jayant Kumar, Adele Salemme, Anna Rita Togna, Luciano Saso, Ferdinando Bruno

**Affiliations:** 1 Department of Natural and Social Sciences, Bowling Green State University-Firelands, Huron, OH 44839, USA; 2 Department of Plastics Engineering and Center for Advanced Materials, University of Massachusetts, Lowell, MA 01854, USA; Ramaswamy_Nagarajan@uml.edu; 3 Department of Physics and Center for Advanced Materials, University of Massachusetts, Lowell, MA 01854, USA; Jayant_Kumar@uml.edu; 4 Department of Physiology and Pharmacology “Vittorio Erspamer”, Sapienza University of Rome, P.le Aldo Moro 5, 00185 Rome, Italy; adelesalemme@libero.it (A.S.); annarita.togna@uniroma1.it (A.R.T.); luciano.saso@uniroma1.it (L.S.); 5 Combat Capabilities Development Command Soldier Center, Natick, MA 01760, USA

**Keywords:** antioxidant activity of phenolic polymers, enzymatic polymerization, polymeric flavonoids, peroxidase catalyzed polymerization, lipase catalyzed polymerization

## Abstract

In recent years, developing potent antioxidants has been a very active area of research. In this context, phenolic compounds have been evaluated for their antioxidant activity. However, the use of phenolic compounds has also been limited by poor antioxidant activity in several in vivo studies. Polymeric phenols have received much attention owing to their potent antioxidant properties and increased stability in aqueous systems. To be truly effective in biological applications, it is important that these polymers be synthesized using benign methods. In this context, enzyme catalyzed synthesis of polymeric phenols has been explored as an environmentally friendly and safer approach. This review summarizes work in enzymatic syntheses of polymers of phenols. Several assays have been developed to determine the antioxidant potency of these polymeric phenols. These assays are discussed in detail along with structure-property relationships. A deeper understanding of factors affecting antioxidant activity would provide an opportunity for the design of versatile, high performing polymers with enhanced antioxidant activity.

## 1. Introduction

Reactive oxygen species (ROS) are known to play a key role in a variety of physiological processes. Production of ROS is continuously balanced by antioxidant (AO) defense mechanisms; however, this delicate balance can be disrupted. Oxidative stress is a physiological condition produced by an imbalance due to excess production of oxidants called ROS and reactive nitrogen species (RNS). When produced in excess, ROS or RNS can interact and damage several cellular membranes and impede normal functioning of the cells. ROS can include a series of oxidants including but not limited to hydrogen peroxide (H_2_O_2_), peroxyl radical (ROO•), singlet oxygen (^1^O_2_) and hydroxyl radical (•OH). The ability to alleviate oxidative stress makes antioxidants an important class of molecules.

Several approaches have been utilized to alleviate oxidative stress including delivery of antioxidants, either small molecules (e.g., vitamin C) or antioxidant enzymes (e.g., superoxide dismutase, catalase) and polymeric antioxidants. Small molecule antioxidants scavenge a variety of ROS and RNS in a non-selective fashion and only in stoichiometric ratios. Hence, large sustained doses are required to observe a significant therapeutic effect. Antioxidant enzymes have been explored as alternatives, but with limited success, since they are prone to pH dependent inactivation and proteolytic cleavage. Enzyme mimics possessing catalytic activities have been designed, however the synthesis protocol often involves multiple steps [1]. While natural antioxidants are still used in many food applications, use of synthetic AO is controversial due to toxicity concerns.

Mixed results from decades of anti-oxidant research has indicated that for an antioxidant therapy to be efficient, three conditions need to be met—(1) The antioxidant must be able to scavenge the correct radicals (2) the radical scavenging must be targeted to the cells during the appropriate time (3) AO must remain active and not degrade for a significant amount of time. In this context the exploration of polymers of phenols as potent and long-lasting AO is relevant. This review summarizes work in the enzymatic syntheses of polymeric phenols for antioxidant applications. Enzymatic methods are discussed in detail to enable design of high-performance polymers. Relevant assays for analyzing AO activity are discussed along with potential pitfalls. AO activity of enzymatically synthesized polymeric phenols and their analogues are summarized and compared with commercial antioxidants.

### Phenolic Antioxidants

**Figure 1:**
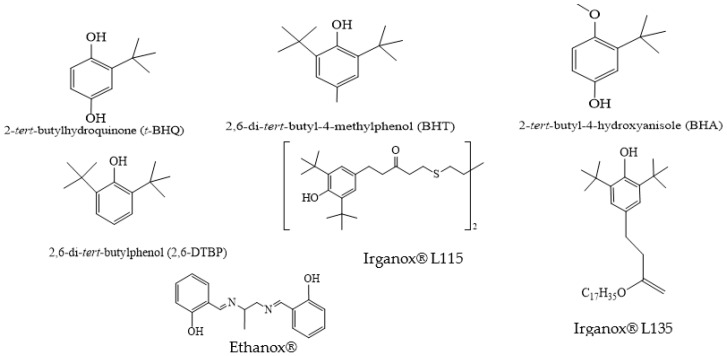
Structures of some commercially used antioxidants.

Phenolic compounds are one group of widely studied compounds useful as antioxidants [2,3,4]. Several hindered phenols used as commercial AO (Figure 1) work by donating their phenolic hydrogen to the generated free radical. Phenoxy radicals generated are resonance stabilized and react with other free radicals. Chemical structure and position of –OH groups influence the AO activity of phenolic compounds. The antioxidant activity of phenolic compounds has been studied in several in vitro studies [5,6].

However, widespread use of phenol-based compounds has been limited due to their low solubility in water and poor in vivo results (low bioavailability and physiological stability, limited absorption and high doses required for antioxidant activity). In organic chemistry, the word “polyphenol” is used to describe the presence of two or more hydroxyl groups on an aromatic compound. However, in publications involving synthesis of polymeric phenols from monomers, the term “polyphenol” is also employed. While this is not completely incorrect, this often leads to confusion. Throughout this paper, polymeric phenols will be used to describe polymers of phenols synthesized from enzyme catalysis.

## 2. Polymer Antioxidants

While small molecule antioxidants mount sufficient defense against many oxidative processes, they have some disadvantages such as leaching which lower the antioxidant performance. This has been compounded with disappointing results from in vivo studies. Interest in use of polymers in antioxidants was spurred by recognition that increasing antioxidant shelf-life in vivo was important to enable these compounds to work effectively. It can be hypothesized that polymeric antioxidants can be a more effective and therapeutic alternative to small molecule antioxidants. Trolox, a well-known antioxidant is a reference standard for total antioxidant potential. While in vitro studies were promising, clinical trials with Trolox have been disappointing. Polymerized Trolox, on the other hand, was found to enzymically degrade to release monomers of active Trolox [7].

Polymers have been used as a drug delivery vehicle for delivering antioxidants to the target site. Encapsulation techniques involve loading of phenolic compounds in colloidal particles prepared using biocompatible polymeric materials [8]. Redox active conductive polymers have been evaluated as antioxidants for their ability for scavenging free radicals. Low oxidation potentials of conducting polymers combined with their tendency to shuttle readily between oxidized or reduced forms [9] can be used to deliver AO to the intended site.

Three main chemical approaches have been used for delivery of AO using the polymer backbone—In the first approach, antioxidant molecules have been functionalized with polymerizable groups [10], which can then be polymerized. As an example, Dziubla et al. have synthesized a new class of poly(β-amino esters) with phenolic antioxidants incorporated in the polymer backbone [11]. The second approach involves derivatization of a polymerizable monomer with an antioxidant molecule [12]. Puoci et al. copolymerized methacrylic acid with ferulic acid to obtain copolymers using radical polymerization [13]. In the third approach, graft polymerization was used to modify the surface properties of the polymer to tailor it for AO applications. Researchers utilized grafting to- and grafting from methods to link antioxidants using chemical synthesis [14] or via melt processing [15]. Phenolic compounds like gallic acid and catechin were grafted onto natural polymers such as gelatin. Hindered phenols have been copolymerized with polyolefins [16].

Phenolic polymers containing multiple AO units can be directly synthesized from corresponding monomers using nucleophilic substitution of halogenated aromatic compounds [17]. However, the method proceeds well only at high reaction temperature and requires cumbersome purification procedures. Compared with these methods, enzyme catalyzed polymerization of phenols has significant advantages including mild reaction temperatures and benign by-products. This book chapter will focus on enzymatic syntheses of polymeric antioxidants.

## 3. Oxidoreductases as Catalysts for Enzymatic Polymerization

Biochemical reactions often need catalysts to proceed at a pace required to sustain life. Interestingly, in many cases, each biochemical reaction is catalyzed by one specific enzyme. Biocatalysis is the use of enzymes as catalysts to enable chemical transformations outside biological systems. With advances in biotechnology, many of these catalysts have been engineered to perform ex vivo under typical reaction conditions encountered in traditional chemical processes. Unlike traditional chemical catalysts, enzymes display distinct properties owing to their complex molecular structure. Enzymes are versatile catalysts when reaction specificity is desired and when environmental restrictions are stringent. Many enzymes, including peroxidases have high turnover number, allowing oxidation of large number of substrate molecules. In addition, when designing polymers for antioxidant applications, it is beneficial to use mild reaction conditions provided by enzyme catalyzed reactions. Hence there have been several research endeavors that have focused on using enzymatic catalysts for the synthesis of polymeric antioxidants based on phenols.

There are more than 2000 human enzymes known [18]. Broadly, Enzymes can be classified into six families—oxidoreductases, transferases, hydrolases, lyases and isomerases, based on the type of chemical reaction they catalyze. The six families are further divided into several sub-classes. Oxidoreductases [19] are versatile proteins known to work under a wide range of reaction conditions. This review will focus on the use of oxidoreductases for the enzymatic polymerization of phenol-based compounds for AO applications. The field of enzymatic polymerization of phenols is dominated by two types of oxidoreductase enzymes, namely peroxidases and laccases

### 3.1. Peroxidases

**Figure 2:**
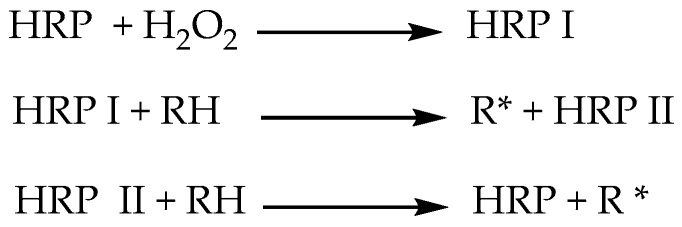
General catalytic scheme for polymerization of phenols catalyzed by peroxidases.

Peroxidases are a sub-class of oxidoreductases which catalyze chemical reactions due to their ability to generate reactive species, which undergo non-enzymatic coupling resulting in the formation of polymers. Two types of peroxidases—Horseradish Peroxidase (HRP) and Soybean Peroxidase (SBP) have been used extensively in the synthesis of polymeric phenols. The mechanism for peroxidase-catalyzed polymerization reactions is well established. As shown in Figure 2, H_2_O_2_ oxidizes the enzyme in the native state to form an important intermediate, HRP-I. Monomer (RH) is oxidized by the action of HRP-I and HRP-II through two sequential one-electron reduction steps via another intermediate HRP-II. As seen in the Figure 2, two moles of radical cations (R*) are generated for every mole of hydrogen peroxide reduced to water. This catalytic cycle continues to progress till oligomers/polymers are formed.

As an oxidant, H_2_O_2_ plays an important role in the peroxidase-initiated phenol polymerization. While periodic addition of the oxidant at regular intervals has been often employed in peroxidase-catalyzed polymerizations, we have found that quick addition of H_2_O_2_ leads to polymeric phenols with higher molecular weights.

However, if the concentration of H_2_O_2_ is too high, polymerization is inhibited due to two principal reasons—(1) H_2_O_2_ is a known inactivator of heme [20,21] and the presence of excess H_2_O_2_ leads to enzyme inactivation. (2) Some peroxidases utilize H_2_O_2_ as an electron donor for the reduction of intermediates formed in the peroxidase cycle. This results in the evolution of oxygen gas, may be described as “catalase-like” since this is similar to the mechanism of action of the enzyme catalase [22]. To keep the concentration of oxidant low, several methods for in-situ generation of H_2_O_2_ have been reported in the literature [23,24,25]. Uyama et al. and others reported a bio-enzymatic system for the polymerization of phenols using peroxidase as catalyst and glucose oxidase as in situ H_2_O_2_ generation catalyst [26,27,28,29].

### 3.2. Laccases

**Figure 3:**
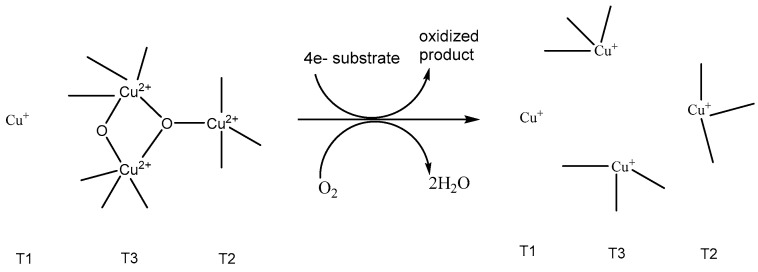
Catalytic scheme for the polymerization of phenols by laccase.

Laccases are copper containing oxidoreductases which are known to catalyze the oxidation of a variety of phenols including substituted phenols and amines while simultaneously reducing molecular oxygen to water. Fungal laccases are the most widely used glycoproteins containing 10–45% by weight of carbohydrates, which contribute towards the stability of the enzyme [30]. The active site of laccase features two Cu atoms each bound to three histidine residues and referred to as the T3 sites. Another third copper exists in a pocket bound to two histidine ligands and referred to as the T2 site and a fourth copper is referred to as the T1 site. Substrate oxidation typically is known to happen at the T1 site, while the T2 and T3 sites form the trinuclear oxygen binding site. The first step involves reduction of Cu^2+^ to Cu^+^ with concurrent oxidation of substrate [31]. The electrons are transferred to the T2/T3 site. Dioxygen is transformed to water through two steps as illustrated in Figure 3**.** In short, the catalytic process involves the four 1 electron oxidation of a substrate with accompanying reduction of dioxygen to water.

## 4. Factors Influencing Peroxidase Catalyzed Polymerization of Phenolic Compounds

Polymerization of phenolic compounds catalyzed by peroxidases can lead to soluble polymers provided appropriate conditions are chosen. This section focuses on four key aspects of the enzymatic reaction—(1) nature and quantity of solvent used to solubilize monomers (2) use of templates to enable greater solubility of monomers in aqueous solutions and promote directed coupling (3) Monomer structure and (4) role of temperature in enzymatic polymerization. All of these factors affect the overall molecular weight of the polymers obtained as well as aqueous solubility of the obtained polymers.

**Figure 4:**
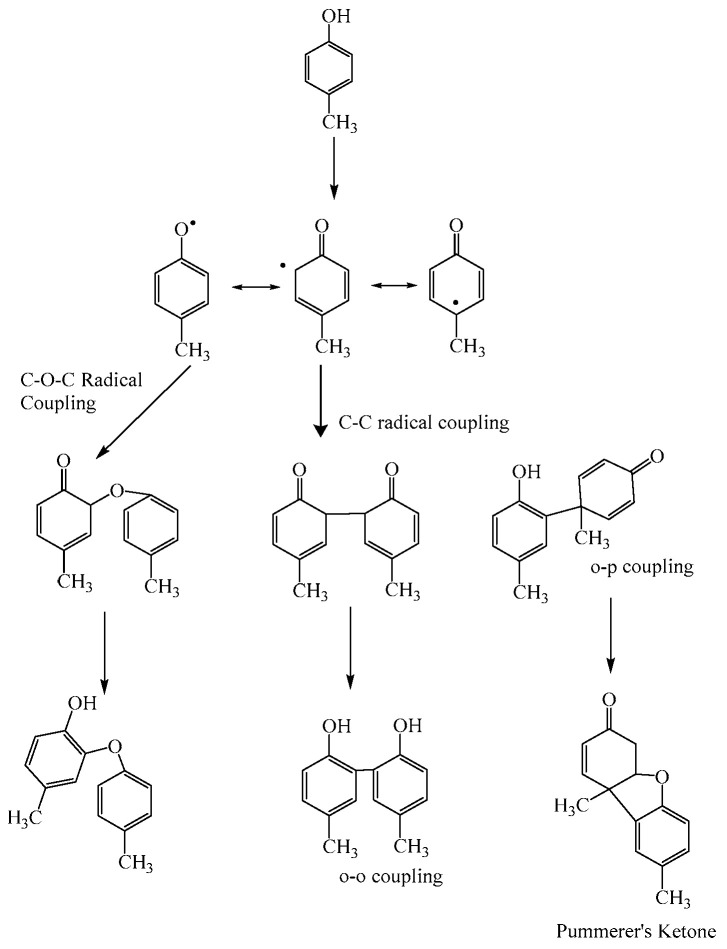
Proposed mechanism for polymerization of 4-methylphenol.

In typical enzymatic reactions, free radicals initially formed from the monomer undergo coupling to produce dimers, trimers and higher oligomers. This process of successive oxidation and coupling of oligomers can lead to the formation of high molecular weight polymers. Sahoo [32] and co-workers used in situ nuclear magnetic resonance (NMR) to probe the nature of coupling reactions that occur in the very early stages of peroxidase catalyzed polymerization. Detailed NMR investigations showed that two kinds of couplings are possible during polymerization of a simple phenol such as *p-*cresol—(a) C−C coupling to form phenylene units and (b) C−O−C coupling to form oxyphenylene units (Figure 4). The authors [33] also report the formation of Pummerer’s ketone as a major side product. The absence of enolic structure in this ketone prevents propagation of polymerization reaction. While the ketone can be removed from the reaction mixture by multiple wash steps, polymer yields are reduced because of ketone formation. The formation of Pummerer’s type ketone must be avoided/suppressed to enable enhanced polymer yield. This is discussed in detail in Section 4.4.

### 4.1. Co-Solvents

The poor solubility of some phenol containing monomers does not allow for the polymerization to be carried out in buffer alone since the reaction terminates with the formation of dimers/trimers that precipitate out of solution. Polymerization in 100% organic solvents is not possible since enzyme activity decreases substantially with increasing concentration of organic solvents. To overcome this, polymerization is either carried out using mixtures of organic solvents and buffer solutions [34,35]. Dordick et al. [36]. reported changes in the molecular weight of poly(phenol) from 1000 to over 26,000 g mol^−1^ depending on concentration of 1,4-dioxane in the polymerization reactions. Mita et al. [37] have studied the role of solvents and their influence on the coupling selectivity. They found that coupling selectivity (C−C versus C−O) can be significantly influenced by changing the hydrophobicity of the monomer. 

Table 1 gives a summary of some of the more commonly used solvent systems for polymerization of different types of phenolic monomers [38]. Proper choice of ratios and concentrations of these solvents can result in control over polydispersity and molecular weight of the polymers.

Besides organic solvents, ionic liquids have been used as co-solvents to aid in the solubility of phenolic monomers. Sgalla et al. [39] noted the formation of dimers using HRP in ionic liquid; 1-butyl-3-methylimidazolium tetrafluoroborate (MIM)[(BF_4_)]) and water. 

Eker et al. reported the formation of higher molecular weight polymers using the same ionic liquid and SBP [49,50]. Besides mixed solvent systems, layer-by-layer (LbL) assembly has also been used to polymerize phenols [51]. The use of organic solvents enabled solubilization and subsequent polymerization of monomeric phenols with limited aqueous solubility. While the use of organic solvents allows formation of processable polymers; it is desirable to have aqueous based systems for biological applications. The use of several water-soluble polymeric templates enabled polymerization of phenols in aqueous systems as outlined in Section 4.2.

### 4.2. Templates

Several studies have shown that templates have an important role to play in oxidoreductase-catalyzed polymerization [52]. In the absence of a template, ill-defined chemical structures have been reported. Since oxidoreductases do not control chain propagation/elongation steps, products formed in absence of a template are often heterogeneous in nature. Templates have been found to effect regioselectivity in polymerization, improve conductivity and molecular weight of polymers and reduce undesirable branched products [53,54,55]. Use of uncharged water-soluble templates like Poly(ethylene glycol) (PEG) enabled polymerization of phenolic compounds to proceed in buffer solutions thus mitigating the need for an organic solvent [56]. It has been suggested that hydrogen bonding between the templates and monomer can confer some selectivity in polymerization favoring high degree of C−C coupling [57,58]. Peng et al. [59] showed that use of carbon nanotubes as a template was found to switch selectivity to oxyphenylene units. In applications where high thermal stability is desired, polyphenols containing higher percentage of oxyphenylene units may be preferred.

Use of micelles as templates for polymerization of phenols has been explored. These include ternary systems such as water/isooctane/bis(2-ethylhexyl) sodium sulfosuccinate or a biphasic system such as water/isooctane [42,60,61,62]. Ultra-high molecular weight polyphenols (M_w_ > 10^6^ Da) were synthesized in the presence of poly(ethylene glycol) (PEG)-poly(propylene glycol) (PPG)-poly(ethylene glycol) (PEG) triblock copolymer (Pluronics^®^) in water [63]. Pluronics are a class of biocompatible polymeric surfactants used widely in medical applications. The authors found that Pluronics with higher PEG content imparted regioselectivity to the reaction, resulting in polymers mainly consisting of phenylene units.

Besides PEG based templates, Xu et al. [64] used dip pen nanolithography to pattern caffeic acid onto a *p*-aminothiophenol-modified gold surface. After patterning, in situ polymerization was done using HRP. Bulk and in situ polymerization yielded different products due to differences in coupling sites. The authors attributed these structural differences to interaction of—COOH groups and primary amine groups in the template. The interaction leads to oriented adsorption of the phenolic monomers and polymerization proceeding with very high phenylene selectivity. Bulk polymerization of the monomer in aqueous conditions lead to a mix of phenylene and oxyphenylene unit and low molecular weight (~1100 Da) products.

Kobayashi et al. performed the enzymatic polymerization of phenolic compounds by using cyclodextrin derivatives (which can be used for solubilizing hydrophobic compounds) [65]. Enzymatic polymerization of coniferyl alcohol using cyclodextrin derivatives was reported to yield greater selectivity in polymerization besides enabling solubility of starting compounds [66].

### 4.3. Monomer Structure

In addition to solvents and templates, selectivity in polymerization reactions has been reported to be influenced by the position and type of functional groups present in the monomer. As an example, polymerization of *p*-hydroxy benzoic acid derivatives was found to proceed producing oxyphenylene units exclusively [67]. Studies done by Mita et al. [68] indicated that hydrophobicity of the substituent controlled the phenylene content of poly(p-substituted phenols). Increase in hydrophobicity increased phenylene content in polymeric phenols. 

Appropriate functionalization/protection of functional groups of monomers prior to polymerization can help overcome unwanted side reactions. Enzymatic polymerization of hydroquinones does not proceed rapidly due to facile oxidation to the corresponding benzoquinones. By monoglycosylation of one of the −OH groups, Wang et al. [69], could polymerize the free −OH group in the hydroquinone and de-protect the glycosidic group post-polymerization. Ritter et al. [70] protected the amine group in 4-amino phenol and then selectively polymerized the resulting monomer leading to higher molecular weight compounds.

### 4.4. Temperature

Influence of temperature on HRP catalyzed polymerization of phenol was studied by Akita et al. [41]. Phenol polymerization was carried out in dioxane-water mixtures at different temperatures from 10 °C to 60 °C. Polymer yield was dependent on the temperature with 80% yield obtained at temperatures between 10 °C to 40 °C. Increase in temperature to 50 and 60 °C decreased polymer yields to 55 and 31% yields respectively. The decrease in the activity of HRP has been attributed to alteration in the secondary and tertiary structure of the enzyme which decreases polymer yields at higher temperatures.

Formation of Pummerer’s ketone, which prevents formation of high molecular weight polymers, was found to be dependent on the temperature. Wu and coworkers [32,71] used in situ NMR to monitor the formation of this Pummerer’s ketone during the polymerization of 4-propylphenol catalyzed by HRP. No ketone formation was observed at temperatures below 293 K. At temperatures, greater than 293 K, increase in intensity of peaks in NMR from the Pummerer’s ketone was observed. The intensity was maximum when the temperature of the reaction was maintained at 313 K for 12 h. Hence use of lower temperature conditions during enzymatic polymerization of phenolic compounds can prevent formation of Pummerer’s ketone and help increase reaction yields.

## 5. Factors Influencing Laccase Catalyzed Polymerization of Phenolic Compounds

### 5.1. Laccase Source

Laccase was first extracted from the Japanese lacquer tree [72]. While laccases from yeast are known, a majority of the characterized laccases have been derived from white-rot fungi. Michaelis constant and catalytic efficiency constant k_cat_ for a number of laccases have been measured [73]. Laccases from different sources have different substrate preferences and variations in the catalytic efficiency of laccases for the same substrate have also been observed. As an example, catalytic efficiencies (k_cat_) for guaiacol oxidation [74] is 150 min^−1^ with laccase *Pleurotus ostreatus* POXC while K_cat_ for the same substrate with laccase *Trametes pubescens* LAP2 is 10,800 min^−1^. Polymerization of derivatives of 4-hydroxybenzoic acid depended on the laccase origin. Laccase from *Pyricularia oryzae* [72] did not catalyze the polymerization of derivatives of hydroxybenzoic acid, while laccase from *Pycnoporus coccineus* was able to catalyze the reaction leading to a product in about 80% yield. 

### 5.2. Reaction Conditions

Formation of phenolic polymers/oligomers from laccase mediated oxidative reactions has been shown to depend on the pH, amount of buffer and nature of organic solvent used during polymerization. Many laccases have been reported to work at around a pH ~5 when phenolic substrates are used [75,76]. Kobayashi et al. reported the synthesis of poly(phenylene oxide) [77] with over 80% yield using 62% of acetone and chloroform as co-solvents. Polymerization of 2,6-dimethylphenol [78] was accomplished using laccase derived from *Pycnoporus coccineous* in a 60:40 dioxane: buffer mixture. The co-solvent used also plays an important role in the solubility and molecular weight of the final polymer. Temperature stabilities of laccases vary considerably, depending on the source of the organism. Laccases are known to increase polymerization rate with increase in temperature up to 37 °C [79].

### 5.3. Use of Redox Mediators

In nature, laccase is known to catalyze lignin biodegradation to generate phenolic compounds. The low redox potential of laccase (0.5–0.8 V vs. NHE) [80,81] allows oxidation of phenolic residues. Other functional groups in lignin have an oxidation potential higher than laccase. It has been hypothesized that low molecular weight easily oxidizable natural metabolites act as initiators. When a substrate is too large to penetrate the enzyme site or has a higher oxidation potential than the enzyme, the presence of redox mediators can facilitate polymerization of these substrates. In a redox mediator assisted polymerization, the mediator first reacts with laccase to form a reactive oxidized intermediate which then oxidizes the substrate of interest. For substrates with higher redox potentials than laccase, researchers have used redox mediators to facilitate oxidation of the substrate. Some of the widely used mediators for laccase catalysis include *N*-hydroxybenzotriazole, 2,2′-azinobis-(3-ethylbenzylthiozoline-6-sulphate), violuric acid and *N*-hydroxy phthalimide. Use of redox mediators for laccase polymerization has also been summarized in review articles [80,81,82].

## 6. Polymerization of Phenols Using Enzyme Catalysis

This section will summarize work done on polymerization of different types of phenolic compounds using enzymatic methods. 

### 6.1. Polymerization of Substituted Phenols

**Figure 5:**
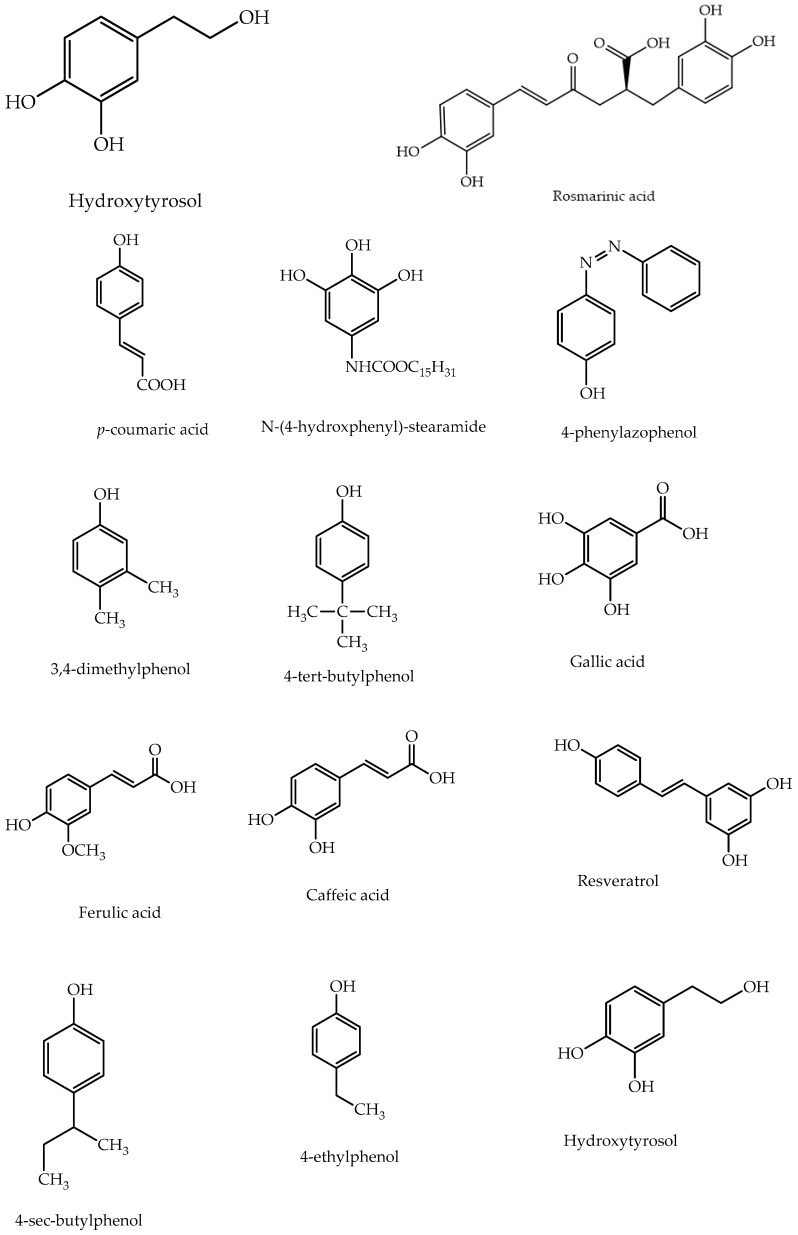

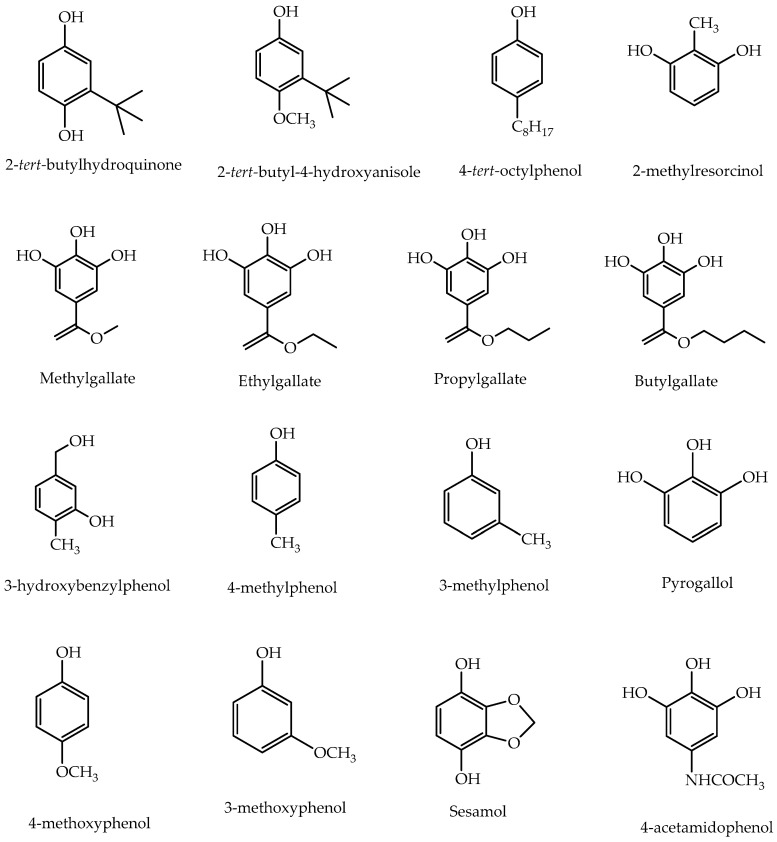
Structures of phenolic monomers polymerized using peroxidases as catalysts to evaluate antioxidant (AO) activity.

Polymerization of a wide range of substituted phenols has been attempted. The structures of these monomers are summarized in Figure 5. Dhawan et al. [83] polymerized substituted phenols using hydrogen peroxide as the oxidant and peroxidases as catalysts. Among the hindered phenol compounds used as AO, 2-*tert-*butylhydroquinone (*t-*BHQ) is an indispensable AO in the food industry. Polymerization of *t-*BHQ was initially attempted using HRP as a catalyst. However, formation of 2-*tert-*butyl-1,4-benzoquinone as a side product prompted the design of a chemo-enzymatic route for synthesis of polymer.

**Figure 6:**
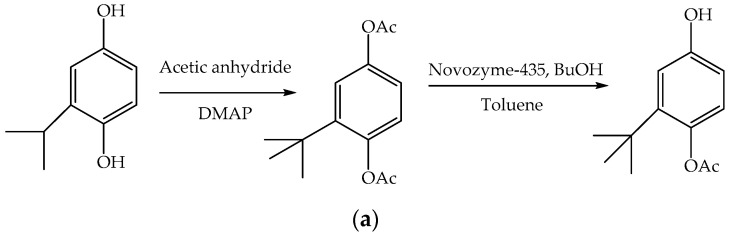

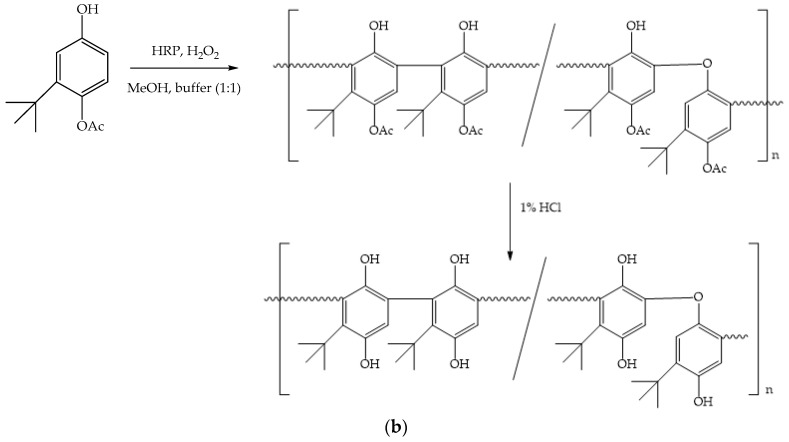
(**a**) Synthesis of 4-acetoxy-3-*tert-*butylphenol; (**b**) Synthesis of polymeric (*t-*BHQ).

In the first step, phenolic hydroxyl groups in *t-*BHQ were first protected by acetylation (Figure 6a,b). Lipase catalyzed reaction of the diacetylated compound in toluene at 37 °C resulted in selective removal of one of the acetyl groups, resulting in the formation of 4-acetoxy-3-*tert-*butylphenol. 

This compound was then enzymatically polymerized using HRP and hydrogen peroxide to form polymers in good yield with enhanced AO activity. Asakura et al. [84] oligomerized several phenolic compounds using HRP (Table 2). Introduction of bulky alkyl groups in the para position prevented the formation of dimers. Introduction of methoxy group in the para position of phenols also increased the molecular weight of the oligomer. Zheng et al. [85] polymerized 4-methoxyphenol in an aqueous micelle system (sodium dodecyl sulfate) with HRP. The polymer obtained was soluble in soluble in acetone, Dimethyl formamide (DMF), Dimethyl sulfoxide (DMSO) and Tetrahydro furan (THF). The number-average molecular weight of poly(4-methoxyphenol) was around 1000 Da and both phenylene and oxyphenylene units were found in the polymer structure.

Pyrogallic acid [47] was enzymatically polymerized in dioxane: buffer solutions using HRP as a catalyst. Polymerization was carried out under different pH conditions and best polymer yield was obtained at a pH of ~7. Different organic solvents were also tested to improve yield of the polymer formed (Table 3). Polymerization in the mixture of 90% buffer and 10% organic solvent (DMF or 1,4-dioxane) yielded polymeric pyrogallol acid in good yields. Increase in organic solvent content to 80% decreased the yield of polymer formed substantially, probably due to loss of enzyme activity. Polymer obtained was found to contain both phenylene and oxyphenylene units. Percentage of organic solvent also influenced molecular weight of the polymer formed (Table 3).

Two naturally occurring phenolic compounds of Rosmarinic acid, commonly found in species of the Boraginaceae and Hydroxytyrosol (−(3,4-dihydroxyphenyl)ethanol) (HDT), found in olive leaves and fruits and extra-virgin olive oil, have been homopolymerized and co-polymerized to yield polymeric compounds. The copolymerization reaction was carried out under ambient atmosphere at approximately 25 °C and was catalyzed by HRP in the presence of hydrogen peroxide in a sodium phosphate buffer and ethanol solution [86,87].

### 6.2. Polymerization of Flavonoids

Flavonoids represent a class of natural occurring phenolic compounds with good antioxidant activity [88,89,90,91,92,93]. Low aqueous solubility, poor bioavailability in vivo and low stability at physiological conditions has prompted enzymatic modification of flavonoids [84].

**Figure 7:**
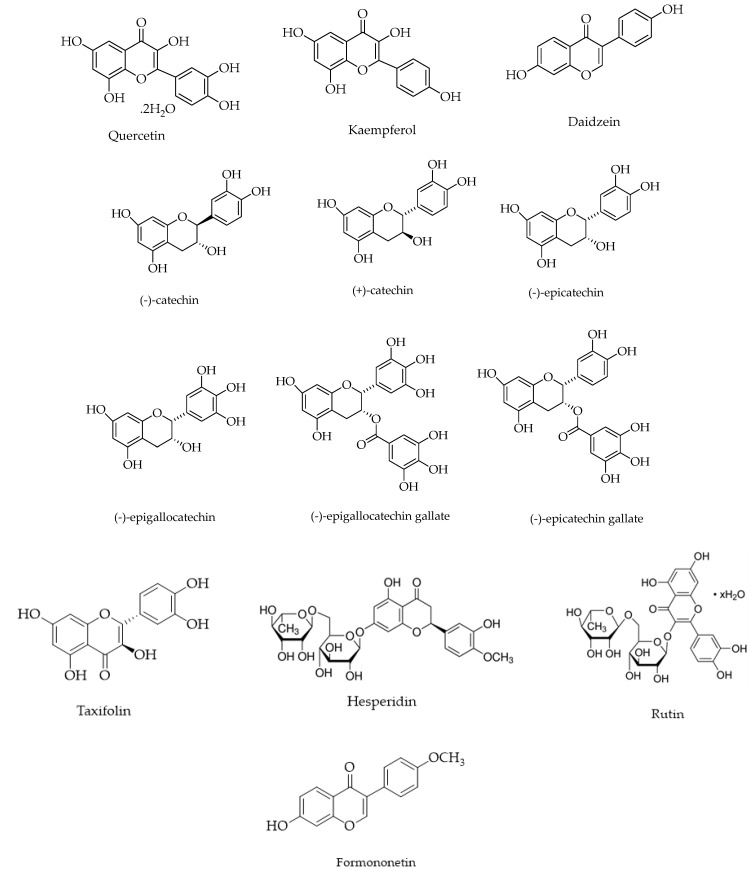
Structures of flavonoids polymerized using enzyme catalysis.

Table 4 and Figure 7 provides a list of flavonoids polymerized using enzymatic catalysis.

Many of these flavonoids are present in green tea including (+)-catechin, (−)-epicatechin, (−)-epigallocatechin, (−)-epicatechin gallate and (−)-epigallocatechin gallate (EGCG). Kobayashi and co-workers have polymerized many green tea catechins using oxidoreductases such as HRP or laccase as catalysts. Rutin and EGCG were enzymatically modified using laccase as catalyst. Poly(rutin) [98] obtained was soluble in water, DMF and DMSO. Oligomeric EGCG obtained was soluble in DMF. (+)-catechin was polymerized in methanol: phosphate buffer (30:70) using HRP as a catalyst [97]. Poly(catechin) obtained was insoluble in water and soluble in DMF, DMSO. Poly(catechin) obtained using laccase catalysis was insoluble in water. To overcome this limitation, Bruno et al. [105] used polymeric templates like PEG to form polymerized catechins that are soluble in water. Polymerization of (−)-epicatechin was performed in phosphate buffer: ethanol mixtures (95:5) to obtain water-soluble polymers [100]. Polymers were separated using HPLC to yield six different fractions with different AO activity. While the fractions exhibited similar absorption spectra, molecular modelling and NMR characterization indicated polymerization to proceed via the A-and C-rings in epicatechin [99]. 

Quercetin [101] and Kaemperol [106] were enzymatically oligomerized in the presence of two enzymes—laccase and tyrosinase. Dihydroquercetin was polymerized using bilirubin oxidase as a catalyst. All polymers obtained show enhanced AO activity.

### 6.3. Enzymatic Oxidation and Grafting of Phenolic Compounds on Biocompatible Polymers

Polysaccharides such as chitosan are biocompatible [108,109] and play an important role in many food systems due to their emulsifying and thickening properties [110]. A variety of polysaccharides including chitosan, hyaluronic acid, alginate, inulin, dextran, pectin and heparin have been used to conjugate flavonoids using chemical coupling, enzyme catalysis and acid catalyzed condensation reactions [111]. These studies have been summarized in Table 5. In many of the grafting processes, the phenolic monomer is oxidized using enzymatic reactions and then undergoes subsequent reactions with a reactive group in the polymer (such as the free amino group in chitosan) through well-established chemical processes such as Michael addition reactions. Among the many polysaccharides, Chitosan, in particular, has been used extensively owing to its excellent biocompatibility [112,113,114]. Laccase has been used to graft ferulic acid and ethyl ferulate to chitosan. Božič and co-workers investigated the effect of pH on laccase catalyzed grafting of gallic and caffeic acids on chitosan [115].

A detailed mechanism for the functionalization has been proposed which involves both electrostatic interactions between ester bonds in gallic acid and amino groups in chitosan. Tannic acid and quercetin have also been grafted on to chitosan using laccase catalysis [123]. Kobayashi and co-workers have conjugated catechin monomer to several polymers like poly(ε-lysine), poly(allyl amine), amine-substituted octahedral silsesquioxane and polymer particles. Conjugation of catechin seems to improve stability and AO activity of the complexes. Overall, the grafting conferred increased AO activity on the polymer while stabilizing the monomeric phenol. Further structural characterization of the polymer-phenol complexes will help in understanding the grafting positions on the polymer chain. The safety profiles of the polymer-phenol complexes have not yet been analyzed. Additional data on safety profiles will help broaden applications of these complexes and enable greater utility of these compounds as AO.

## 7. Methods to Evaluate Antioxidant Activity

Given the plethora of phenolic compounds known, it is important to develop and utilize methods for systematic evaluation of antioxidant activity of these compounds. Antioxidant potency of phenolic monomers and polymers is evaluated using well-established assays discussed in this section. Many of these assays use reaction kinetics to measure the rate of quenching of a given reactive species by the antioxidant.

The ability of AO to scavenge free radicals is assessed by two important parameters (1) rate of scavenging (2) number of free radicals scavenged per antioxidant molecule. Huang et al. [127] classified AO assays based on the chemical reactions involved into—(1) hydrogen atom transfer (HAT) reaction-based assays and (2) single electron transfer (ET) reaction-based assays. HAT based assays measure the ability of an antioxidant to quench free radicals by hydrogen donation. On the other hand, the ET-based assays utilize redox reactions to measure the AO activity. An oxidant in the reaction is reduced by the antioxidant, accompanied by change in color which can be measured spectrophotometrically. AO potency of phenolics have been analyzed in the literature using ET and HAT based assays. Given the plethora of assays which are used to evaluate the antioxidant activity, it is more meaningful for researchers to use more than one essay to determine antioxidant activity.

### 7.1. DPPH Assay

**Figure 8:**
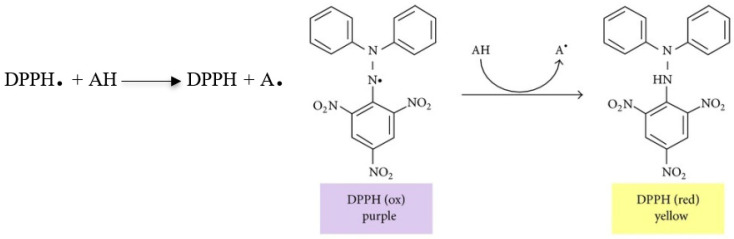
Structure of 2,2-Diphenyl-1-picrylhydrazyl (DPPH) in oxidized and reduced forms [128].

2,2-Diphenyl-1-picrylhydrazyl (DPPH) is a commercially available radical stable in methanol solution. In the radical form, DPPH has an absorption maximum at 515 nm but upon reduction by an antioxidant (AH), this absorption decreases (Figure 8). In this ET based assay, absorbance of the sample is monitored at 515 nm for 30 min or till the time when the absorbance becomes stable. DPPH radical scavenging activity (%) is calculated using the formula—(1 − (A_sample_/A_control_) × 100). Sometimes, scavenging abilities are also reported as IC_50_ or EC_50;_ which is defined as the concentration that causes a decrease in the initial DPPH concentration by 50%. While the DPPH assay is simple, the DPPH radical is stable and does not resemble the reactive and short-lived free radicals seen in many biological processes.

### 7.2. ABTS Assay

2,2-Azinobis-(3-ethylbenzothiazole-6-sulphonate) (ABTS) is oxidized using oxidants such as potassium persulfate. In this ET-based assay the radical cation, ABTS^•+^ is generated from ABTS. Ferryl myoglobin radical is also used in some assays to generate the ABTS radical cation. In a typical assay procedure, ABTS can react with the oxidants/radicals at room temperature to form the radical cation. The formation of ABTS^•+^ is minimized in the presence of antioxidants. Unlike, DPPH• which is soluble in organic solvents, ABTS^•+^ is soluble in water and alcohols and its absorption at 734 nm is used to monitor AO potency. Since absorption is measured at 734nm, interference from absorption of other compounds is minimized in this assay. Phenolic compounds/polymers with high molecular mass and/or multiple –OH groups have the tendency to aggregate in solution. In such instances, these compounds are less able to diffuse to the active sites of the sterically hindered ABTS radical which will in turn limit the accessibility of this reagent during electron transfer. As a result, the electron transfer rate constants for hindered donor-acceptor complexes are lower and can vary with solvent polarity [129].

### 7.3. β-Carotene-Linoleic Assay

The oxidation of linoleic acid (LH) is known to generate peroxyl free radicals. The free radicals formed oxidize *β*-carotene and this oxidation is minimized in the presence of AO. In the first step, an emulsion is prepared by dissolving *β*-carotene, LH and Tween, a surfactant in Chloroform. Chloroform is evaporated under nitrogen and the mixture is shaken vigorously with water to form an emulsion with absorption maximum at 470 nm. The oxygen dissolved in water oxidizes LH. β-carotene reacts with radicals formed as a result of linoleic acid oxidation. The rate of *β*-carotene bleaching is slowed down when an antioxidant is present through transfer of hydrogen atom from the AO. The decrease in bleaching of the carotenoid is a function of both the antioxidant concentration and its potency.

### 7.4. Ferric Reducing Ability of Plasma (FRAP) Assay

Benzie et al. [130,131,132] developed a method to determine the reducing ability of plasma as a function of its antioxidant power. While the assay was initially used to determine radical scavenging ability of plasma, it was also later applied to other natural compounds. A ferric salt, Fe(III)(TPTZ)_2_Cl_3_ (TPTZ = 2,4,6-tripyridyl-*s*-triazine), is used as an oxidant. The reaction is carried out at low pH conditions (pH ~4–5), when the Fe (III) is reduced to Fe (II) by electrons donated from the antioxidant. Formation of Fe (II) complex is accompanied by the appearance of an intense blue color with an absorption maximum of 593 nm. Change in absorbance is usually monitored for 4–8 min, making this a rapid screening assay. Presence of metal chelators in the reaction mixture that bind to Fe (III) can alter assay results [127]. Pulido et al. [133] reported that the FRAP reaction assay time of 4 min does not work with several monomeric phenol compounds like caffeic acid, tannic acid and quercetin. Longer reaction times are required when using the FRAP assay for testing the AO power of phenol-based compounds.

### 7.5. Xanthine Oxidase (XO) Assay

Xanthine oxidase is a protein that catalyzes the oxidation of hypoxanthine to xanthine and generates superoxide in the process. Antioxidant activity toward superoxide radical can be determined using xanthine-xanthine oxidase system as the radical source and probes such as ferricytochrome *c* or nitrobluetetrazolium (NBT). The reduction of both probes leads to the formation of ferrocytochrome *c* or formazan. In the presence of an antioxidant, the reduction of ferricytochrome *c*/NBT is inhibited. While the reduction of both the probes is mediated by xanthine oxidase, flavonoids directly inhibit xanthine oxidase. This can sometimes lead to an overestimation of antioxidant capabilities.

### 7.6. Suppression of Low Density Lipoprotein (LDL) Oxidation

Several studies have indicated that antioxidants mitigate problems associated with lipid oxidation. Lipid hydroperoxides are the primary products in the early stage of LDL oxidation. To analyze the effect of antioxidants on LDL oxidation, LDL is first labeled with Diphenyl-1-pyrenylphosphine (DPPP). DPPP reacts stoichiometrically with the lipid hydroperoxide to form diphenyl-1-pyrenylphosphine oxide (DPPPdO), which is fluorescent. The free radical generator 2,2′-azobis(2-amidinopropane) dihydrochloride (AAPH) is used to initiate oxidation of LDL. In a typical assay, DPPP labeled LDL is pre-incubated with a sample of antioxidant, prior to initiation of oxidation by addition of AAPH. In the absence of antioxidant, fluorescence intensity increases with time due to formation of hydroperoxides. However, in the presence of an antioxidant, decrease in the fluorescent intensity is observed which is attributed to scavenging of free radicals in the hydroperoxides by antioxidants.

### 7.7. Oxygen Radical Absorption Capacity (ORAC) Assay

This assay measures antioxidant activity by hydrogen atom transfer. The compound Fluorescein is used as a fluorescent probe in the ORAC assay. The assay measures the decrease in fluorescence of the probe over time due to peroxyl-radical formation. The peroxyl radical is formed from the breakdown of AAPH. Trolox (6-Hydroxy-2,5,7,8-tetramethylchroman-2-carboxylic acid), is used as a positive control; since it inhibits fluorescein decay in a dose dependent manner. The fluorescence signal is measured over 30 min by excitation at 485 nm. The concentration of antioxidant in the test sample is proportional to the fluorescence intensity. This is measured by comparing the net area under the curve to that of Trolox. The Unites States Department of Agriculture (USDA) removed its ORAC database “due to mounting evidence that the values indicating antioxidant capacity have no relevance to the effects of specific bioactive compounds, including polyphenols on human health.” In addition, USDA also stated that results from ORAC assays are often misused by dietary supplement manufacturing companies.

## 8. Antioxidant Activity of Monomeric and Polymeric Phenols

### 8.1. Antioxidant Activity of Substituted Phenols

The AO of a number of polymerized phenols as measured by the beta-carotene assay is summarized in Table 6 [83]. As seen in the table, all polymeric phenols reported an enhanced AO compared to their monomeric analogues. Besides the beta-carotene assay, several oligomeric phenols prevent the autoxidation of tetralin hydroperoxide. Asakura and co-workers [84] showed that phenolic oligomers exhibited a better AO activity than the corresponding monomers. The authors calculated the degree of free hydroxyl groups remaining in all oligomers. The number of free hydroxyl groups correlated with the observed AO activity with oligomers with higher free hydroxyl groups exhibiting better AO activity. The AO activity of phenolic polymers is also summarized in Table S1 presented in the Supplementary Materials.

### 8.2. Antioxidant Activity of Monomeric and Polymeric Flavonoids

Flavonoids exhibit pleiotropic mechanisms in radical scavenging. Flavonoids inhibit enzymes involved in superoxide anion formation such as xanthine oxidase [134,135,136] and enzymes responsible for reactive oxygen species generation such as lipoxygenase, microsomal monooxygenase and cyclooxygenase [137]. Flavonoid compounds are reported to be scavengers of the superoxide and peroxyl radicals [3,138]; however, this has also been questioned and contradicted in the literature [139,140]. These compounds also inhibit, glutathione *S*-transferase and NADH oxidase [141]. In addition, flavonoids are shown to be effective inhibit lipid peroxidation [142,143,144], inhibit LDL oxidation induced by copper ions [145,146]. The AO potency was found to depend on the pH (high activity was seen between pH 6–12) and nature of metal ions present [147]. While presence of copper (II) ions increased the antioxidant activity, presence of iron (II) ions decreased the radical scavenging activity.

**Figure 9:**
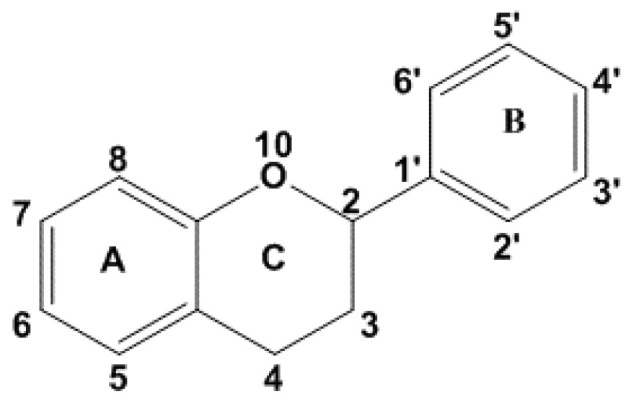
Basic structure of a flavonoid.

Figure 9 depicts the basic structure for the flavonoids. Structure-activity relationships between flavonoid structure and its relation to the observed AO ability has been studied [148]. Three major structural features in the flavonoid are important for observing high AO potency. (1) Presence of a *o-*dihydroxy structure in the B ring confers greater scavenging ability. Metal chelating ability is attributed to the presence of catechol moiety. (2) Presence of a 2,3-double bond in conjugation with a carbonyl group in C-4 and (3) presence of a –OH group on C-3 and C-5 along with a 4-oxo function in the A and C rings is required for good radical scavenging potential

Among the flavonoids, the AO activity of catechins found in green tea have been studied. Polymerized catechins are known to possess enhanced AO activity against the superoxide anion, XO when compared to a (+)-catechin monomer. Polymerized epicatechin [99] was shown to exhibit enhanced AO activity in ORAC assay. Since enzymatic polymerization often produces multiple products of varying chain length, polymeric epicatechin obtained using enzyme catalysis was separated using HPLC analysis. Among the six fractions obtained from HPLC analysis, fraction 6, which was shown to be non-polar with higher content of hydroxyl groups showed highest AO activity. Polymerized epicatechin was also effective in inhibiting flaxseed oil oxidation.

Oligomerized epigallocatechin gallate (EGCG) exhibited enhanced radical scavenging activity against superoxide anion and was shown to inhibit xanthine oxidase. Oligomeric EGCG exhibited better AO activity than Trolox, Butylated hydroxytoluene (BHT) and allopurinol. In contrast, the EGCG monomer did not show any significant AO activity. Polymerized rutin showed good superoxide scavenging activity and inhibition effects on LDL. Rutin monomer was shown to exhibit pro-oxidant activity under the same conditions.

Table 7 summarize AO activity of number of flavonoid monomers and polymers. The AO activity of commercial AO (BHT and BHT) are also included for comparison.

### 8.3. Antioxidant Activity of Grafted Polymers

Nine different flavonoids—Catechin (C), epicatechin (EC), Epigallocatechin (EG), Epigallocatechin gallate (EGG), Fisetin, Quercetin, Rutin, Hesperedin and Daidzein were grafted on chitosan [118]. Observed AO activity was found to depend on the type of the flavonoid and the percentage of flavonoid grafted onto chitosan. The authors do not provide results on the AO activity of flavonoids alone, tested under similar reaction conditions.

In the catechin family, C, EC, EG and EGG grafted on chitosan show enhancement of antioxidant activity with the highest value reported EG, which is attributed to the 3′,4′,5′-trihydroxyl groups on B-ring. The spatial arrangement of substituents on the flavonoid ring, the configuration and number of hydroxyl groups determine efficacy [149]. Since EG has three –OH groups in the B ring, high antioxidant activity is expected. By the same analogy, presence of galloyl moiety in EGG should increase the observed AO activity. However, the lower AO activity is being attributed to coupling reactions and steric hindrance. Quercetin and Fisetin are flavonols with a 2-3-double-bond conjugated with 4-oxo moiety. This conjugation allows for delocalization of electrons and stabilization of phenoxyl radical. Structurally, the only difference in Quercetin and fisetin is the absence of a 5-OH group in the fisetin’s structure. This difference is crucial and can explain the differences in AO activity of these flavones grafted on to chitosan.

Rutin and hesperidin showed insignificant enhancement in antioxidant activity. This is due to lack of planarity, absence of a 3-OH group (which is crucial for radical scavenging) and steric hindrance when grafted on chitosan. A small enhancement in the AO activity is noted for Daidzein. AO power of flavonoids linked grafted on chitosan is summarized in Table 8. Flavonoids have also been conjugated with other polymers such as poly(allylamine), poly-ε-lysine and polyhedral oligomeric silsesquioxane. Conjugation, in general seems to improve AO activity of these polymers (Table S2 in Supplementary Materials).

## 9. Conclusions and Future Directions

Traditional methods used for the synthesis of antioxidant polymers are often laborious and have lower antioxidants moieties per unit weight, thus reducing the overall AO performance of these macromolecular complexes. Since many naturally occurring phenolic compounds have good AO profiles, these molecules can serve as excellent starting materials for the synthesis of polymeric antioxidants. Enzyme catalyzed polymerization enables synthesis of polymeric phenols under environmentally friendly conditions. Oxidoreductases have been widely employed for polymerization of a wide range of phenolic compounds. Enzymatic polymerization augments the AO activity of phenolic compounds while increasing stability and solubility in aqueous media. Further, molecular weight of the resulting phenolic polymers can be judiciously tailored using homo/co-polymerization methods to achieve optimal solubility and AO activity. Polymerization has been reported to increase the number of electron-donating aromatic hydroxy groups, enables electron delocalization and stabilizes the phenolic radical. This has been correlated with enhancements in AO activity observed on polymerization. For example, polymerization of 3-hydroxybenzyl phenol yields polymers with over 350% increase in AO activity on polymerization using the beta carotene method [150]. Phenolic compounds have been grafted onto biocompatible polymers such as chitosan, poly(allylamine), poly(ε-lysine). As with direct polymerization, AO activity has been shown to be either preserved or enhanced upon grafting and conjugation.

Commercial polymeric phenol-based antioxidants marketed by Polnox^®^ are currently being used as antioxidants in a wide range of materials and consumer products including elastomers, lubricants and biofuels [151]. Polymeric antioxidants outperform small molecule commercial phenolic based compounds, confirming that polymerization enhances AO performance.

There continues to be significant interest in developing antioxidants based on phenolic compounds for biological applications. While the literature is replete with reports on the health benefits from antioxidants, indiscriminate antioxidant supplementation may cause problems since reactive oxygen and nitrogen species perform vital functions. Excessive removal of oxidant species can potentially distort cell signaling pathways and increase chronic disease risks [152]. Hence preserving the balance of oxidants and antioxidants in the body is crucial to health.

As new phenolic polymers are designed, research is required to develop in antioxidant assays which address inherent limitations associated with some of the in vitro assays. Currently, antioxidant potency for phenolics in vitro are evaluated using reactant radicals that do not mimic biologically relevant free radicals and hence are not predictive of in vivo behavior. In addition, biologically important radicals, such as (•OH, ROO•) are often used in excessive quantities which is not a true reflection of physiological conditions. An ideal assay to probe antioxidant activity according to Apak et al. [153] should (1) work at a pH range close to physiological conditions; (2) use stable probes that work well in aqueous and organic phases; (3) use chromophores with a well-defined absorption in the visible region to avoid interferences from other compounds; (4) use chemical reagents with high reduction potential to oxidize a variety of compounds. (5) exclude strong chelators and reductants when using transition metal ion coordination complexes. (6) exclude redox cycling of transition metal coordination complexes with hydrogen peroxide or oxygen. To accurately predict AO activity, specific information on the type of oxidation products that are inhibited by phenolics and their relationship to biological source(s) must be probed. Further, more specific assays involving products that actually cause damage in biological systems need to be developed. More studies on the bioavailability of polymerized forms of phenolic compounds are also needed.

## Figures and Tables

**Table 1:** Common solvent systems used in polymerization of phenol containing monomers.

Solvent System	Monomer	Enzyme	References
Phosphate buffer and DMF (80:20)	*p*-cresol and sucrose	Invertase, glucose oxidase and SBP	[40]
1,4 dioxane, DMF or methanol with water	Phenol	HRP	[41]
Water and dioxane (85:15)	Ethylphenol	HRP	[42]
Succinate buffer and dioxane	Phenol	Glucose oxidase & HRP	[26]
Phosphate buffer and 1.4-dioxane	Catechol	HRP	[43]
Acetate buffer and dioxane	Phenol	HRP	[36]
Buffer and isooctane	*p-*ethylphenol	HRP	[44]
Dioctylsodiumsulfosuccinate (AOT) and isooctane; AOT, isooctane and CHCl_3_	*p-*ethylphenol	HRP	[45]
AOT and isooctane	*o*-chlorophenol	Laccase	[46]
Different solvent systems	Pyrogallic acid	HRP	[47]
HEPES buffer & Langmuir Blodgett films	Phenol	HRP	[48]

**Table 2:** Molecular weight of polymers obtained by polymerization of substituted phenols catalyzed by Horseradish Peroxidase (HRP) [84].

Compound	M_n_	M_w_/M_n_
Oligo (4-methylphenol)	796	1.45
Oligo (4-ethylphenol)	770	1.38
Oligo (4-sec butylphenol)	803	1.28
Oligo (4-tert-butylphenol)	790	1.23
Oligo (3-methylphenol)	558	1.15
Oligo (3-methoxyphenol)	532	1.24
Oligo (4-methoxyphenol)	1747	3.17
Oligo (3,4-dimethylphenol)	732	1.41

**Table 3:** Effect of organic solvent on the molecular weight of polymerized (pyrogallic acid) [47] and effect of organic solvent on the molecular weight of polymerized(pyrogallic acid).

**Organic Solvent (%)**	**Percent Yield of Polymer**				
	**DMF**	**DMSO**	**1,4-Dioxane**	**Ethanol**	**Methanol**
5	26	60	63	8	6
10	38	66	70	20	19
20	47	63	60	33	34
40	59	54	55	56	53
50	65	42	35	35	41
60	44	37	12	23	30
**Organic Solvent and Percentages Used**				**M_n_ (×10^3^)**	**M_w_ (×10^3^)**
DMSO (10%)				2.3	3.8
1,4-Dioxane (10%)				2.5	4.3
DMF (50%)				3.1	4.9
Ethanol (40%)				3.3	10.5
Methanol (40%)				3.9	13.5

**Table 4:** Flavonoids polymerized using peroxidases and laccases.

Monomer	Synthesis Method	References
(+)-catechin hydrate	Enzyme catalyzed using HRP and laccase	[94,95,96]
Epigallocatechin gallate	Enzymatic modification using laccase	[97]
Rutin	Enzymatic modification using laccase	[98]
(−)-epicatechin	Enzymatic modification using HRP	[99,100]
Quercetin	Laccase and Tyrosinase	[101]
Resveratrol	Chemoenzymatic route involving laccase	[102]
Resveratrol	Oligomerization using HRP	[103]
Gallic acid	Laccase	[104]
Daidzein, rutin, Quercetin, Formononetin	SBP and HRP	[94]
(−)-catechin	Oligomerization using HRP	[105]
Taxifolin and kaempferol	Oligomerization using laccase	[106]
Arbutin and gentisate	HRP	[107]

**Table 5:** Flavonoids conjugated/grafted on biocompatible polymers.

Polymer	Phenolic Monomer	Enzyme	References
Poly(ε-lysine)	Catechin	Laccase	[116]
Gelatin	Catechin	Laccase	[117]
Chitosan	Catechin, epigallocatechin gallate, epigallocatechin, epicatechin, Quercetin, Fisetin, Rutin hydrate, Hesperidin, Daidzein	Tyrosinase	[118,119]
Chitosan	Catechin	Laccase	[120]
Chitosan	Quercetin, Rutin, Naringin, Hesperdin	Chloroperoxidase	[121]
Chitosan	Ferulic acid and ethyl ferulate	Laccase	[122]
Chitosan	Quercetin and tannic acid	Laccase	[123]
Silsesquioxane	Catechin	HRP	[124]
Poly(allylamine)	Catechin	Laccase	[125]
Acrylic polymers	Catechin	Laccase	[126]

**Table 6:** Antioxidant potency of polymerized phenols and monomers using the β-carotene assay [83].

Compound	% Antioxidant Activity Determined by β-Carotene Assay	
	Monomer	Polymer
2-*tert*-butylhydroquinone (t-BHQ)	5.3	13.2
2-*tert*-butyl-4-hydroxyanisole (BHA)	44.2	53.2
Sesamol	13.4	34.2
2-methylresorcinol	2.3	6.6
Methylgallate	4.7	19.4
Ethylgallate	4.4	17.2
propylgallate	4.2	16.1
Butylgallate	4.1	16.4
4-acetamidophenol	3.9	9.4
*N-*(4-hydroxyphenyl)-steramide	3.1	8.4
*4-tert-*octylphenol	1.8	7.5
*p-*coumaric acid	6.8	8.8
3-hydroxybenzylphenol	1.0	5.0
4-methylphenol	0.9	5.0
4-phenylazophenol	5.4	25.5

**Table 7:** Summary of the AO activity of polymerized and monomeric flavonoids ^a^ Reactive Oxygen Species (ROS) was generated by the oxygenation of dihydrorhodamine 123. Presence of antioxidants decreased fluorescence.

Compound	Xanthine Oxidase (XO) (µM)	LDL	Superoxide Scavenging Activity; IC_50_ (µM)	ORAC	Comments
(+)-Catechin [95]	34% at 200 uM [95], IC_50_ > 200 [95]				
Poly(catechin) [95]	90% at 200 uM.		92.7 ± 8.7		
EGCG [97]	Very little to no inhibition reported		59.0 ± 4.7		
Oligomeric EGCG [97]	100% inhibition at 50 µM		12.7 ± 1.4		
Rutin [98]	N/A				Monomer rutin was shown to exhibit prooxidant activity against XO
Poly(rutin) [98]	300 µM (100% inhibition)	400µM			
Poly(epicatechin) [99]				Different polymeric epicatechin fractions inhibited at concentrations between 5 to 14 ppb.	Monomer epicatechin does not inhibit fluorescein at concentrations used in the assay
Polymeric quercetin [101] using laccase a			More than 50% inhibition at 60 µg/mL; over 100% inhibition at lower concentration.		Proxidant effect seen at 180 µg/mL
Polymeric quercetin [101] using Tyrosinase ^a^			More than 100% inhibition at conc. over 86 µg/mL		Proxidant effect seen at >200 µg/mL
Polymeric Kaempferol using Tyrosinase [101] a			More than 100% inhibition at conc. over 85 µg/mL		Proxidant effect seen at 255 and 85 µg/mL
Polymeric [106] Kaempferol using laccase ^a^			More than 50% inhibition at 71 µg/mL; over 100% inhibition at lower concentration.		Proxidant effect seen at 180 µg/mL

**Table 8:** Summary of AO activity for flavonoids grafted on chitosan.

Compounds Grafted on Chitosan	DPPH (% Inhibition)	ABTS (% Inhibition)	Superoxide Anion Scavenging (% Inhibition)
Catechin [118]	87.40 ± 0.03	8.49 ± 0.03	36.89 ± 0.05
Epicatechin [118]	88.46 ± 0.04	20.97 ± 0.03	1.07 ± 0.03
Epigallocatechin [118]	No enhancement seen	20.03 ±0.03	73.89 ± 0.08
Epigallocatechin gallate [118]	53.98 ± 0.03	26.75 ± 0.03	35.06 ± 0.03
Fisetin [118]	41.10 ± 0.10	3.41	32.87 ± 0.30
Quercetin [118]	24.18 ± 0.08	25.50 ± 0.03	38.89 ± 0.03
Rutin [118]	6.55 ± 0.07	17.95 ± 0.03	No enhancement seen
Hesperedin [118]	7.93 ± 0.03	21.29 ± 0.03	48.89 ± 0.02
Daidzein [118]	2.45 ± 0.01	26.92 ± 0.03	19.67 ± 0.04
Tannic acid [118]	91% inhibition at pH 4.5; 96% inhibition at pH 6.5		
Quercetin [118]	95% inhibition; 96% inhibition at pH 6.5		
Ferulic acid [122]	EC_50_ (mg/mL) = 0.52 ± 0.04	EC_50_ (mg/mL) = 0.20 ± 0.02	
Ethyl ferulate [122]	EC_50 (_mg/mL) = 1.50 ± 0.04	EC_50_ (mg/mL) _=_ 0.66 ± 0.04	

## Supplementary Materials

The following are available online at https://www.mdpi.com/2073-4360/12/8/1646/s1, Table S1: Summary of the AO activity of polymerized phenols. Table S2: Antioxidant activity for catechin conjugates with polymeric matrices.
