# Supplementary material for: Antioxidant Activity of Synthetic Polymers of Phenolic Compounds
Source: Polymers (Basel). 2020 Jul 24;12(8):1646. doi: 10.3390/polym12081646 (PMC7464737; doi:10.3390/polym12081646)
Supplement: Supplementary file 1 [file polymers-12-01646-s001.pdf]

Table S1: Summary of the AO activity of polymerized phenols

Compound	DPPH (% inhibition)	ABTS (% inhibition)	FRAP	Comments
Oligo(taxifolin) [107]		IC_{50} = 4µg/ml		
Poly(caffeic acid) [101]		100% inhibition		actual conc. of polymer in assay not reported.
Polymerized(4-methoxyphenol) [85]	IC_{50} = 7.08 mg/ml	IC_{50} = 4.62 mg/ml		
Polymerized (pyrogallic acid) [47]	1.4-5.8 mg L^{-1}	0.92-4.7mg/L	4.1 -22.4 mmol Fe^{2+} g^{-1}	No data for AO activity of pyrogallol monomer.
Arbutin – gentisate conjugate [108]	143µM			Conjugate showed lower AO activity than gallic acid.
Butylated hydroxyanisole (BHA)	IC_{50} = 3.3 mg L^{-1}	IC_{50} = 1.9 mg L^{-1}	14.6 mmol Fe^{2+} g^{-1}	
Butylated hyroxytoluene (BHT)	IC_{50} = 13.5 mg L^{-1}	IC_{50} = 3.8 mg L^{-1}	11.6 mmol Fe^{2+} g^{-1}	

Table 2: Antioxidant activity for catechin conjugates with polymeric matrices.

Compound	DPPH and ABTS	Xanthine Oxidase (XO) (μM)	LDL	Superoxide anion scavenging	Comments
Catechin conjugated with poly(allylamine) [125]			Inhibition of LDL was dependent on the concentration of catechin-conjugate used.		
Poly(ε -lysine)-catechin conjugate [116]		~80% inhibition at 300μM; no inhibition shown by poly(ε-lysine)			XO inhibition shown by conjugate is reported to be higher than polymerized catechin synthesized using enzyme catalysis.
Catechin conjugate with polyhedral oligomeric silsesquioxane [124]		60% inhibition at 300μM.		100% inhibition at 150 μM. concentration of the conjugate	No scavenging observed with oligomeric silsesquioxane.
Grafting catechin on gelatin [117]			Inhibition is concentration dependent and is observed between 20-80 μM	50% inhibition at catechin concentration of 4-5 μM	No activity was observed from gelatin
Catechin immobilized on amine containing porous polymer particles using laccase [126]	90% inhibition of DPPH for particle conc. of 100 mg; 90% inhibition of ABTS for particle conc. of 10mg				
BHT				>>200	
Trolox				155 ± 7.8	
